# Regulation of neural stem cells by innervating neurons

**DOI:** 10.1111/jnc.16287

**Published:** 2025-01-07

**Authors:** Nicole Leanne Dittmann, Lauren Chen, Anastassia Voronova

**Affiliations:** ^1^ Neurosciences and Mental Health Institute University of Alberta Edmonton Alberta Canada; ^2^ Department of Medical Genetics, Faculty of Medicine & Dentistry University of Alberta Edmonton Alberta Canada; ^3^ Department of Cell Biology, Faculty of Medicine & Dentistry University of Alberta Edmonton Alberta Canada; ^4^ Faculty of Medicine & Dentistry, MS Centre University of Alberta Edmonton Alberta Canada; ^5^ Women and Children's Health Research Institute University of Alberta Edmonton Alberta Canada

**Keywords:** cell differentiation, innervation, neural stem cell, neurogenesis, olfactory bulb, SVZ

## Abstract

The adult central nervous system (CNS) hosts several niches, in which the neural stem and precursor cells (NPCs) reside. The subventricular zone (SVZ) lines the lateral brain ventricles and the subgranular zone (SGZ) is located in the dentate gyrus of the hippocampus. SVZ and SGZ NPCs replace neurons and glia in the homeostatic as well as diseased or injured states. Recently, NPCs have been found to express neurotransmitter receptors, respond to electrical stimulation and interact with neurons, suggesting that neuron‐NPC communication is an emerging critical regulator of NPC biology. In this review, we discuss reports that demonstrate neuronal innervation and control of the neurogenic niches. We discuss the role of innervating neurons in regulating NPC fates, such as activation, proliferation, and differentiation. Our review focuses primarily on the innervation of the SVZ niche by the following neuronal types: glutamatergic, GABAergic projection and interneurons, cholinergic, dopaminergic, serotonergic, neuropeptidergic, nitrergic, and noradrenergic. We also discuss the origins of SVZ niche innervating neurons, such as striatum, cortex, basal ganglia, raphe nuclei, substantia nigra and ventral tegmental area, hypothalamus, and locus coeruleus. Our review highlights the various roles of innervating neurons in SVZ NPC fates in a spatiotemporal manner and emphasizes a need for future investigation into the impact of neuronal innervation on NPC gliogenesis.

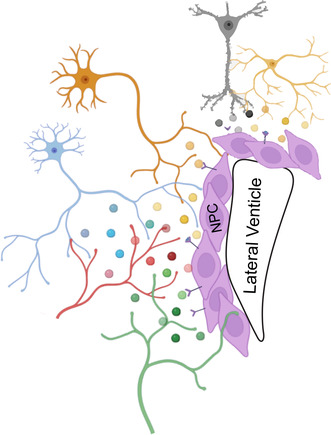

## Graphical abstract

To “build” the brain, neural stem and precursor cells (NPCs) first proliferate, and then differentiate into neurons and glia (astrocytes and oligodendrocytes) in a strict spatiotemporal manner. “Maintenance” of adult brain function requires a continuous generation of neurons and glia via adult NPCs, which are also required for replacement of damaged or lost central nervous system cells in response to injury. Neuron‐NPC communication is an emerging critical regulator of brain development and regeneration. In this review, we discuss neuronal innervation of neurogenic niches throughout life and the neurochemical mechanisms of NPC fate regulation.

**Abbreviations**
5,7‐DHT: 5,7‐dihyroxytryptamine
5‐HT: serotonin
6‐ODHA: 6‐hydroxydopamine
AAV: adeno‐associated virus
ACC: anterior cingulate cortex
Ach: Acetylcholine
AcTub: Acetylated tubulin
AD: Alzheimer's disease
Ank3: Ankyrin 3 or Ankyrin G
BrdU: bromodeoxyuridine
CA3: cornu Ammonis 3
CaMKIIa: calcium/calmodulin‐dependent protein kinase IIa
Casp3: cleaved caspase 3
CD44: cluster of differentiation 44
ChAT: choline acetyltransferase
Chr2: channelrhodopsin
CLCF1: cardiotrophin‐like cytokine factor 1
CNO: clozapine N‐oxide
CNS: central nervous system
CNTF: ciliary neurotrophic factor
CR: calretinin
CT‐1: cardiotropin‐1
D1L: dopamine family D1‐like receptor
D2L: dopamine family D2‐like receptor
DA: dopamine
DAT: dopamine transporter
Dcx: doublecortin
DiI: 1,1′‐dioctadecyl‐3,3,3′,3′‐tetramethylindocarbocyanine perchlorate
Drd2: Dopamine receptor D2
DREADDS: designer receptors exclusively activated by designer drugs
E: embryonic day
EGFR: epidermal growth factor receptor
Emx1: empty spiracles homeobox 1
EPSC: excitatory postsynaptic currents
eYFP: enhance yellow fluorescent protein
FACS: Fluorescent‐activated cell sorting
FGFR: fibroblast growth factor receptor
FITC: fluorescein isothiocyanate
FKN: fractalkine; CX3CL1
FRET: fluorescence energy transfer
GABA: γ‐aminobutyric acid
Gas6: growth arrest specific 6
GDNF: glial‐derived neurotropic factor
GFAP: glial fibrillary acidic protein
GFP: green fluorescent protein
GLAST: glutamate aspartate transporter
Gsh2: γ‐glutamylcysteine synthetase 2
Iba1: ionized calcium‐binding adapter molecule 1
IFNγ: interferon gamma
IPs: intermediate progenitors
IPSC: inhibitory postsynaptic currents
JAK: janus kinase
Ki67: antigen kiel 67
LC: locus coeruleus
LGE: lateral ganglionic eminence
LIF: leukemia inhibitory factor
MAO: monoamine oxidases
Mash1: mammalian achaete‐scute homolog 1 (also known as ASCL1)
MCAO: middle cerebral artery occlusion
MGE: medial ganglionic eminence
Mip1β: macrophage inflammatory protein 1β
MPTP: 1‐methyl‐4‐phenyl‐1,2,3,6‐tetrahydropyridine
MrgA1: mas‐related gene A1
NA: noradrenergic
NADPH: nicotinamide adenine dinucleotide phosphate hydrogen
Nestin: neuroepithelial stem cell protein
NeuN: neuron specific nuclear protein
NF: neurofilament
Nkx2.1: NK2 homeobox 1
NO: nitric oxide
NOS1: nitric oxide synthase 1
NPC: neural stem and precursor cells
NpHR: halorhodopsin
NPY: neuropeptide Y
NPY/AgRP: neuropeptide Y/Arginine‐related peptide
Nrtn: neurturin
NT‐1: netrin 1
OB: olfactory bulb
Olig2: oligodendrocyte transcription factor 2
OPC: oligodendrocyte precursor cells
Otx2: orthodenticle homeobox 2
P: postnatal day
Pax6: paired box 6
PCNA: proliferating cell nuclear antigen
PCPA: parachlorophenylalanine
PD: Parkinson's disease
PDGFRβ: platelet‐derived growth factor receptor β
POMC: pro‐opiomelanocortin
PSA‐NCAM: polysialylated neuronal cell adhesion molecule
RMS: rostral migratory stream
Satb2: special AT‐rich sequence‐binding protein 2
SGZ: subgranular zone
SNc: substantia nigra
Sox2: sex‐determining region Y protein‐box transcription factor 2
STAT: signal transducers and activators of transcription
SuM: supramamillary nucleus
SVZ: subventricular zone
TAPs: transit amplifying cells
Tbr2: T‐box brain protein 2
TH: tyrosine hydroxylase
TTX: tetrodotoxin
TUNEL: terminal deoxynucleotidyl transferase biotin‐dUTP nick end labeling
Vcam‐1: vascular cell adhesion molecule 1
Vgat: vesicular GABA transporter
Vglut2: vesicular glutamate transporter 2
VTA: ventral tegmental area
WGA: wheat germ agglutinin
WT: wild‐type
YFP: yellow fluorescent protein
Zic1: zinc finger protein ZIC 1

## 1 INTRODUCTION

The adult mammalian brain maintains several neurogenic niches, including the subventricular zone (SVZ) lining the ventricles, the subgranular zone (SGZ) in the hippocampus (Chaker et al., 2016; Obernier & Alvarez‐Buylla, 2019) as well as the more recently discovered hypothalamic neurogenic niche (Bartkowska et al., 2023; Sharif et al., 2021). Neural stem and precursor cells (NPCs) located in these neurogenic niches share markers with astrocytes, such as glial fibrillary acidic protein (GFAP), and glutamate aspartate transporter (GLAST) and express unique markers such as vascular cell adhesion molecule 1 (Vcam‐1), neuroepithelial stem cell protein (Nestin), and sex‐determining region Y protein‐box transcription factor 2 (Sox2). While NPCs and astrocytes share many markers, these cells are distinct and perform different functions (Velloso et al., 2022). Key properties of stem cells are self‐renewal and multipotency. Adult NPCs maintain their pools throughout life via sustained proliferation and can differentiate into neurons and glia, thereby contributing to the renewal of central nervous system (CNS) cells and physiological processes such as memory, cognition, and sensory regulation (Chaker et al., 2016; Deng et al., 2010; Obernier & Alvarez‐Buylla, 2019; Silvas‐Baltazar et al., 2023). During differentiation, NPCs exit quiescence, become activated, proliferate, and commit to doublecortin‐positive–positive (Dcx)+, polysialylated neuronal cell adhesion molecule‐positive (PSA‐NCAM) + neuroblasts, which then differentiate into neurons that express neuron‐specific nuclear protein (NeuN). Alternately, NPCs give rise to glia (David‐Bercholz et al., 2021; Obernier & Alvarez‐Buylla, 2019). The ability of NPCs to activate declines with age (Audesse & Webb, 2020; Kalamakis et al., 2019; Merson, 2020).

SGZ NPCs generate immature neurons, which migrate radially to first populate the inner granule cell layer of the dentate gyrus before extending dendrites to the molecular layer and projecting axons into the cornu Ammonis 3 (CA3) region, where they regulate neuronal networks (Akers et al., 2014; Deng et al., 2010; Snyder et al., 2011). While hippocampal neurogenesis in children is well‐accepted, hippocampal neurogenesis in adult humans is currently under debate (Simard et al., 2024; Tosoni et al., 2023). Nevertheless, some reports demonstrate adult hippocampal neurogenesis in humans, which is reduced in patients with Alzheimer's disease (AD) (Moreno‐Jiménez et al., 2019; Tiwari et al., 2023). This reduction in adult hippocampal neurogenesis is thought to contribute to a cognitive decline in AD. In addition to neurons, SGZ NPCs also form astrocytes but not oligodendrocytes under physiological conditions (Obernier & Alvarez‐Buylla, 2019; Steiner et al., 2004).

SVZ NPCs represent the largest pool of NPCs in the mouse and human brain. Under normal physiological conditions, murine adult SVZ NPCs primarily contribute to olfactory bulb (OB) neurogenesis by committing to intermediate progenitors (IPs) that express mammalian achaete‐scute homolog 1 (Mash1; also known as Ascl1) and neuroblasts, which migrate tangentially along the rostral migratory stream (RMS), eventually reaching the OB (Chaker et al., 2016; Lois et al., 1996). Once they reach the OB, they migrate radially into various OB neuronal layers and differentiate into inhibitory neurons (interneurons). Similar to SGZ neurogenesis, SVZ‐mediated OB neurogenesis in humans is debated, but it is generally accepted to occur at least in childhood (Bressan & Saghatelyan, 2021). Although SVZ NPCs predominantly generate OB interneurons, they also give rise to both oligodendrocytes and astrocytes. For example, NPCs in the dorsal SVZ give rise to oligodendrocytes in the adult homeostatic and demyelinated mouse brain (Gonzalez‐Perez & Alvarez‐Buylla, 2011; Menn et al., 2006; Nait‐Oumesmar et al., 1999; Ortega et al., 2013; Picard‐Riera et al., 2002; Xing et al., 2014). Moreover, SVZ NPCs can contribute to astrogliogenesis as well as to formation of cortical neurons in stroke (Chaker et al., 2016).

NPCs in the SVZ and SGZ are a heterogeneous population and vary in their response to physiological inputs, proliferative state, and generation of differentiated cells (Borrett et al., 2020; Borrett et al., 2022). For example, depending on their location in either the dorsal or ventral SVZ, NPCs can generate different OB interneurons and/or oligodendroglial cells (Bond & Song, 2021; Delgado et al., 2021; Merkle et al., 2014). Single‐cell RNA‐sequencing of the SVZ niche further confirms this regional heterogeneity in a spatially restricted manner (Llorens‐Bobadilla et al., 2015; Mizrak et al., 2019). These regional differences in SVZ NPC properties are at least in part because of their corresponding developmental origins. Postnatal NPCs in mice are specified by embryonic day (E)15.5, when some of the NPCs are set aside to divide more slowly and are destined to become adult NPCs (Bond & Song, 2021; Fuentealba et al., 2015; Furutachi et al., 2015; Yuzwa et al., 2017). The lateral SVZ in the adult mammalian brain originates from the embryonic lateral ganglionic eminence (LGE), defined by γ‐glutamylcysteine synthetase 2‐positive (Gsh2) + cells (Young et al., 2007), whereas the septal SVZ NPCs originates from septal progenitors, which express zinc finger protein ZIC1 (Fuentealba et al., 2015). The dorsal and dorsolateral wall NPCs are derived from empty spiracles homeobox 1‐positive (Emx1) + cortical progenitors, while the ventral SVZ NPCs arise from NK2 homeobox 1 (Nkx2.1) + medial ganglionic eminence progenitors (MGE) (Fuentealba et al., 2015).

While OPCs are well known to form *bone fide* synapses with excitatory and inhibitory neurons, which regulate OPC proliferation, survival, differentiation, and myelination (reviewed in (Maas & Angulo, 2021; Moura et al., 2022; Paukert & Bergles, 2006; Taylor & Monje, 2023)), much less is known about the ability of SVZ and SGZ NPCs to form synapses and to communicate with neurons. What is known is that NPCs respond to electrical activity in the brain or external electrical stimulation. For example, seizures increase hippocampal neurogenesis (Parent et al., 1997; Pastor‐Alonso et al., 2024). Moreover, SGZ NPCs can contribute to seizures and epilepsy by aberrant generation of granule cells in the dentate gyrus (Danzer, 2019). Adult SVZ NPCs increase proliferation, migration and neurogenesis in vivo, as well as oligodendrogenesis in vitro, when exposed to clinically relevant electric fields (Babona‐Pilipos et al., 2011; Cao et al., 2013; Dong et al., 2019; Iwasa et al., 2019; Sefton et al., 2020). Moreover, SVZ NPCs express neurotransmitter receptors (Borrett et al., 2022; Dittmann et al., 2023), and pharmacological manipulation of neurotransmitters and their receptors affects NPC fates (Bovetti et al., 2011; Young et al., 2011). For example, γ‐aminobutyric acid (GABA) reduces proliferation of both postnatal and adult SVZ NPCs and neuroblasts and promotes elongation of newborn neuron dendrites (Eduardo et al., 2006; Laurent et al., 2003; Liu et al., 2005). Finally, NPCs interact with neurons or axons present in the NPC niche (discussed below).

**FIGURE 1:**
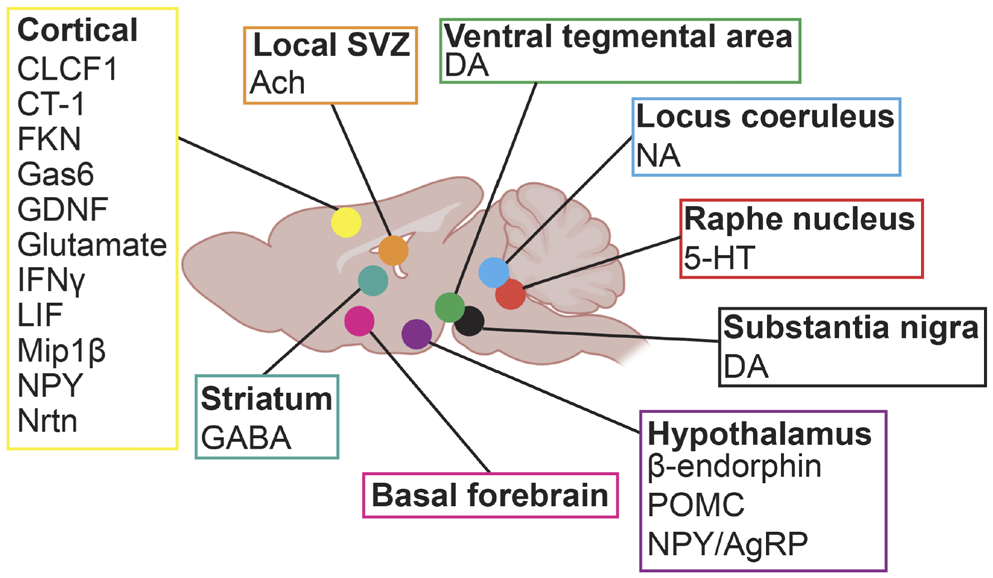
Summary of sources and identified neurochemicals regulating SVZ and/or SGZ NPCs. Regions highlighted are in the mouse brain. This figure was generated using BioRender and Adobe Illustrator. 5HT, serotonin; Ach, acetylcholine; CLCF1, cardiotrophin like cytokine factor 1; CT‐1, cardiotropin‐1; DA, dopamine; FKN, fractalkine, CX3CL1; GABA, γ‐aminobutyric acid; Gas6, growth arrest specific 6; GDNF, glial derived neurotropic factor; IFNγ, interferon gamma; LIF, leukemia inhibitory factor; Mip1β, macrophage inflammatory protein 1β; NA, noradrenaline; NO, nitric oxide; NPC, neural stem and precursor cells; NPY, neuropeptide Y; NPY/AgRP, neuropeptide Y/Arginine related peptide; Nrtn, neurturin; POMC, pro‐opiomelanocortin; SGZ, subgranular zone; SVZ, subventricular zone.

**FIGURE 2:**
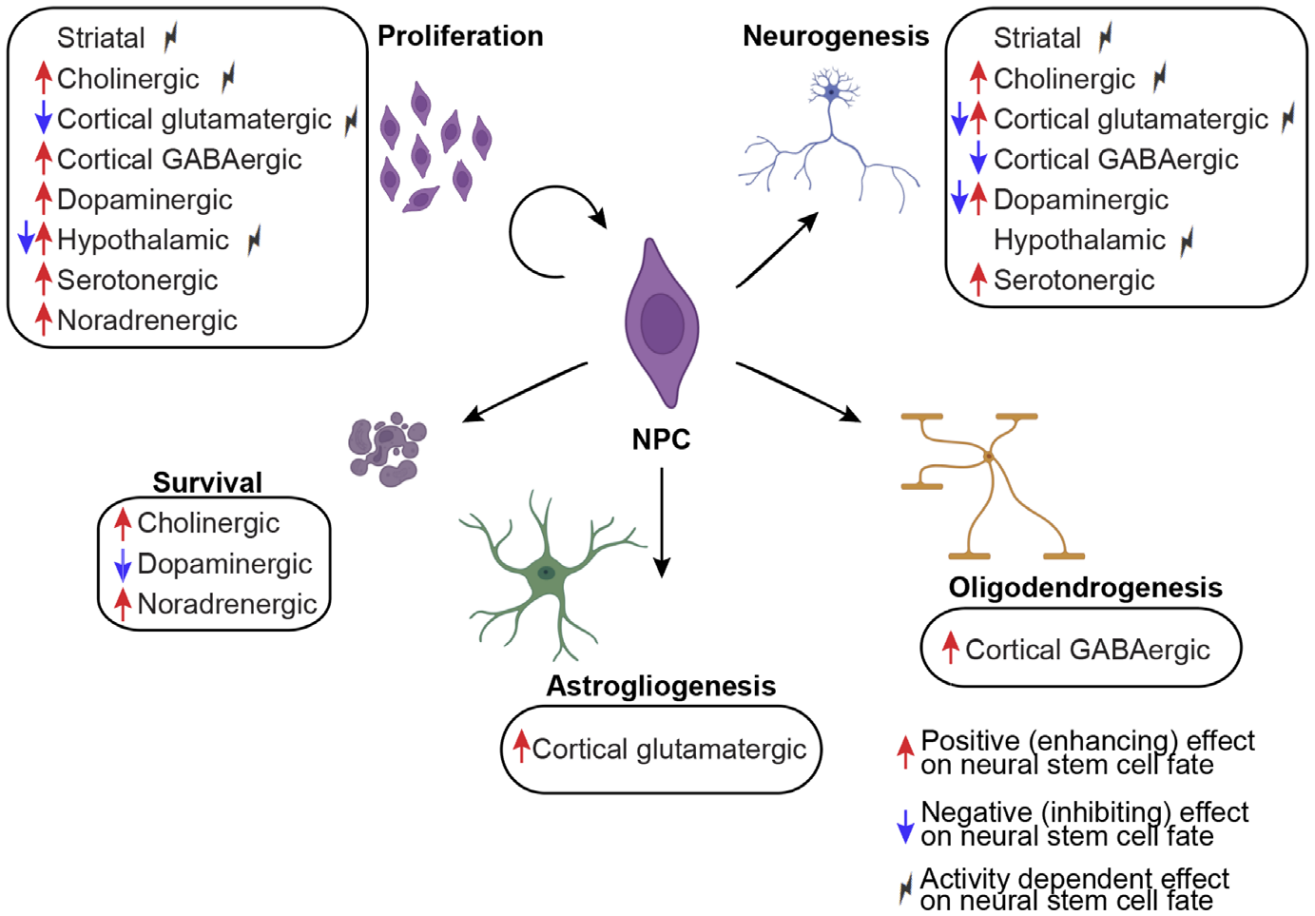
Summary of effects of neuronal innervation on SVZ and/or SGZ NPC fates. Red arrows indicate a positive/enhancing effect, blue arrows indicate negative/inhibiting effect on NPC fates. For example, if a lesion results in reduced NPC proliferation, it is interpreted that the normal role of neurons in this area is to support proliferation (red arrow). Results because of neuronal activity modulation (e.g. optogenetic or chemogenetic activation/silencing) are noted with a lightening bolt. Please see relevant sections text for more details. This figure was generated using BioRender and Adobe Illustrator. GABA, γ‐aminobutyric acid; NPC, neural stem and precursor cells; SVZ, subventricular zone; SGZ, subgranular zone.

This review will summarize the current knowledge on NPC fate regulation by neighboring neurons or innervating axons (Table 1; Figures 1 and 2). We will primarily focus on the SVZ niche during brain development, homeostasis, and injury or disease. We will give examples of how the axonal innervation regulates SGZ NPCs when applicable.

**TABLE 1:** Summary of sources and subtypes of SVZ and/or SGZ innervating neurons and their effect on NPC fates.

Innervating neurons	Neurotransmitters/neurochemicals	Neurogenic niche	Age	Species	Role in NPC fates	References
Striatal	GABA NO Ach	SVZ	Postnatal & Adult	Mice	Activity‐dependent regulation of calcium waves in SVZ NPCs in an adult homeostatic brain	Young et al. (2014)
	Unknown				Activity‐dependent regulation of neurogenesis in a stroke model	He et al. (2016)
Basal forebrain	Ach	SVZ	Adult	Mice	Cholinergic regulation of OB neurogenesis or newborn neuron survival through lesions and pharmacological inhibition	Cooper‐Kuhn et al. (2004), Kaneko et al. (2006)
Local SVZ					Activity‐dependent regulation of proliferation and neurogenesis in an adult homeostatic brain	Naffaa et al. (2023), Paez‐Gonzalez et al. (2014)
Diagonal band		SGZ			Activity‐dependent regulation of proliferation in an adult homeostatic brain	Chen et al. (2024)
Cortical	CLCF1, CT‐1, FKN, GABA, Gas6, GDNF, Glutamate, IFNγ, LIF, Mip1β, NPY, Nrtn	SVZ	Embryonic	Mice	Presumably tonic secretion of neurochemicals promoting neuro‐ and gliogenesis in the developing cortex	Barnabé‐Heider et al. (2005), Voronova et al. (2017), Yuzwa et al. (2016)
	Glutamate		Adult		Cortical regulation of neuro‐ and gliogenesis or proliferation through lesions and activity‐dependent manipulations	Naffaa et al. (2023), Ota et al. (2023)
Substantia Nigra	DA	SVZ	Adult	Mice & Rats	Dopaminergic regulation of SVZ proliferation, neurogenesis and survival in adult homeostatic and pharmacologically lesioned brain	Baker et al. (2004), Höglinger et al. (2004), Peng et al. (2008), Sui et al. (2012), Winner et al. (2006)
				Primates		Freundlieb et al. (2006)
Ventral tegmental area				Mice & Rats	Dopaminergic regulation of SVZ proliferation in pharmacological and genetic ablation models	Höglinger et al. (2014), Lennington et al. (2011)
Hypothalamic	POMC β‐endorphin	SVZ	Adult	Mice	Activity‐dependent regulation of ventral SVZ proliferation and neurogenesis, modulated by feeding behavior in adult homeostatic mice	Paul et al. (2017)
	NPY/AgRP Orexin			Rats	Orexigenic regulation of dorsal SVZ NPC proliferation in adult homeostatic and pharmacological lesioned rats	Arias‐Carrion et al. (2018)
	GABA Glutamate	SGZ		Mice	Activity‐dependent regulation of SGZ NPC proliferation and neurogenesis in adult homeostatic mice	Li et al. (2022)
Raphe nucleus	5HT	SVZ/SGZ	Adult	Mice & Rats	Serotonergic regulation of SVZ/SGZ proliferation and neurogenesis in adult homeostatic and pharmacologically lesioned mice	Brezun and Daszuta (1999), Brezun and Daszuta (2000), Tong et al. (2014)
Locus coeruleus	NA	SVZ	Embryonic	Rats	Noradrenergic regulation of cell survival and proliferation in embryonic brain explants	Popovik and Haynes (2000)
		OB	Postnatal & Adult	Mice, Rats & Sheep	Noradrenergic regulation of NPC apoptosis, proliferation, and modulation of OB behaviors	Bauer et al. (2003), Brennan et al. (1998), Levy (1990), Sullivan et al. (1992), Veyrac et al. (2005)

*Note*: Neurotransmitters and neurochemicals listed characterize the innervating neurons. For list of neurochemicals affecting NPC fates, please see Figure 1 and text for more details.

Abbreviations: 5HT, serotonin; Ach, acetylcholine; CLCF1, cardiotrophin‐like cytokine factor 1; CT‐1, cardiotropin‐1; DA, dopamine; FKN, fractalkine; CX3CL1; GABA, γ‐aminobutyric acid; Gas6, growth arrest specific 6; GDNF, glial‐derived neurotropic factor; IFNγ, interferon gamma; LIF, leukemia inhibitory factor; Mip1β, macrophage inflammatory protein 1β; NA, noradrenaline; NO, nitric oxide; NPC, neural stem and precursor cells; NPY, neuropeptide Y; NPY/AgRP, neuropeptide Y/Arginine‐related peptide; Nrtn, neurturin; OB, olfactory bulb; POMC, pro‐opiomelanocortin; SGZ, subgranular zone; SVZ, subventricular zone.

### 1.1 Regulation of NPCs by striatal GABAergic and nitrergic neurons

The striatum is a collection of deep brain nuclei that regulate the initiation and inhibition of movement, as well as emotion and cognition (Lanciego et al., 2012). The striatum is located directly adjacent to the lateral SVZ (Figure 1), and uniquely supports SVZ NPC differentiation. Specifically, Magnusson and colleagues have elegantly demonstrated that the transplantation of SVZ and SGZ NPCs into the striatum, but not the cortex, supports their commitment to Dcx + neuroblasts and differentiation into mature NeuN+ neurons (Magnusson et al., 2020).

A vast majority (~78%) of striatal neuronal projections next to or in the SVZ niche are in close proximity to SVZ NPCs and neuroblasts (Young et al., 2014). Patch clamp recordings of the striatal neurons extending axons or dendrites into SVZ identified them as medium spiny GABAergic projection neurons (77.5%), with a minor population of pacemaker aspiny neurons (20%), and cholinergic neurons (2.5%) (Young et al., 2014) (Figure 1). Another report has additionally identified nitric oxide (NO) striatal neuron projections that express NADPH‐diaphorase (marker for nitrergic neurons) next to the PSA‐NCAM+ neuroblasts in the SVZ (Moreno‐López et al., 2000) (Figure 1). Stimulation of striatal neurons projecting into the SVZ results in increased calcium currents recorded in SVZ NPCs (Young et al., 2014). Notably, increases in the frequency of calcium transients and the number of SVZ cells responding to striatal neuron stimulation were blocked with both tetrodotoxin (TTX), a pan‐inhibitor of action potentials, and bicuculine, which blocks GABA_A_ receptors (Young et al., 2014). Previous work has found that GABA regulates SVZ NPC and neuroblast proliferation and migration via the GABA_A_ receptor (Bolteus & Bordey, 2004; Laurent et al., 2003; Liu et al., 2005; Stewart et al., 2002). However, unlike previous studies, which suggested GABA signaling in the SVZ acts as a paracrine factor from tonic release, Young and colleagues suggested striatal neurons can regulate adult NPCs in an activity dependent manner through the GABA_A_ receptor (Young et al., 2014) (Table 1; Figure 2). The effect of activity‐dependent GABA release from striatal neuron projections on SVZ NPC fates, such as proliferation and differentiation, remains unknown.

The effect of striatal neuron activity on adult SVZ neurogenesis was investigated by a separate group in a murine stroke model via middle cerebral artery occlusion (MCAO) (He et al., 2016). Optogenetic inhibition of striatal neuron activity 4–7 days after MCAO significantly improved functional outcomes, such as reduced brain atrophy and improved motor performance. Conversely, activation of striatal neuron activity resulted in greater brain atrophy, although there was no difference in motor performance compared with the control group (He et al., 2016). The authors also assessed netrin‐1 (NT‐1) expression, an axon guidance factor that is thought to be beneficial in stroke recovery (He et al., 2013; Lu et al., 2016). As with the behavioral experiments, the reduction in striatal neuron activity increased NT‐1 abundance, with the opposite occurring with increased striatal neuron activity (He et al., 2016). Finally, activation of striatal neurons reduced, while inhibition of striatal neurons increased the number of bromodeoxyuridine (BrdU) + Dcx + proliferating neuroblasts and Nestin signal intensity in the SVZ compared with control. With regard to the peri‐infarct region, striatal neuron inhibition led to a higher number of newborn BrdU+NeuN+ cells compared with control (He et al., 2016) (Table 1; Figure 2). Together, these experiments suggest inhibition of striatal neuron activity promotes neurogenesis and recovery following ischemic stroke (He et al., 2016) (Table 1; Figure 2). Although He and colleagues did not investigate whether the effect of striatal neuron activity on neurogenesis was because of GABA, their finding of reduced neuroblast proliferation is consistent with existing work on GABA's effect on SVZ cells (Bolteus & Bordey, 2004; Laurent et al., 2003; Stewart et al., 2002).

### 1.2 Regulation of NPCs by cholinergic neurons originating from basal forebrain and SVZ niche

Cholinergic neurons, which are characterized by the expression of neurotransmitter acetylcholine (Ach), have multiple discrete sources in the brain and project diffusely to various brain regions (Dautan et al., 2016). The largest source of cholinergic neurons is in the striatum, with additional populations in the basal forebrain (e.g., diagonal band and septum), brainstem, hypothalamus, and OB (Ahmed et al., 2019; Hebb & Silver, 1961; Macintosh, 1941; Woolf & Butcher, 1982). However, Paez‐Gonzalez and colleagues demonstrated that choline acetyltransferase (ChAT) + cholinergic neuronal projections in the SVZ niche originate not from the known cholinergic brain nuclei, but from a novel population located in the SVZ niche itself (Paez‐Gonzalez et al., 2014) (Figure 1). While the developmental origin of these local ChAT+ neurons was not established, they can be lineage traced in cells that express dopamine D2 receptor (Drd2) (Paez‐Gonzalez et al., 2014), which is expressed in striatal neurons as early as E17 (Daws & Konradi, 2011).

Knockout of *Chat* or Ankyrin 3 (Ank3 or Ankyrin G), a critical regulator of axonal initial segment assembly and therefore axon potential generation, in Drd2+ striatal neurons results in a lack of Ach release from local SVZ cholinergic neurons and concomitant SVZ neurogenesis defects (Paez‐Gonzalez et al., 2014). These defects included reduced Ki67+ proliferating cells, Mash1+ transit amplifying cells (TAPs), and Dcx + neuroblasts (Paez‐Gonzalez et al., 2014). Optogenetic silencing of local SVZ cholinergic neurons corroborated these results (Paez‐Gonzalez et al., 2014). In agreement, optogenetic activation of cholinergic neurons in the SVZ resulted in increased numbers of proliferating NPCs and total numbers of Mash1+ TAPs and Dcx + neuroblasts in the SVZ (Paez‐Gonzalez et al., 2014) (Table 1; Figure 2). These findings were extended in a separate report, which showed that increased Ach signaling achieved via long‐term (4‐week) administration of a cholinesterase inhibitor donepezil increases cell survival in the OB, as evidenced by a greater number of BrdU+ cells compared with saline‐treated animals (Kaneko et al., 2006) (Table 1; Figure 2). Importantly, modulation of distal striatal cholinergic neuronal activity in vivo does not perturb SVZ NPC fates (Paez‐Gonzalez et al., 2014). Moreover, SVZ cell fates are not altered in *Nkx2.1Cre;Chat* ^*fl/fl*^ mice, which lack Ach release from distant striatal, but not local SVZ, cholinergic neurons (Paez‐Gonzalez et al., 2014). Together, this provides compelling evidence that the local cholinergic neurons in the SVZ niche regulate NPC fates in an activity‐dependent manner (Table 1; Figure 2). With regard to the SGZ, cholinergic regulation of SGZ NPC fates was reviewed elsewhere (Madrid et al., 2021). Moreover, it was recently discovered that cholinergic neurons originating from the diagonal band innervate the SGZ and regulate SGZ NPC proliferation in an activity‐dependent manner by forming a network with local granule cells (Chen et al., 2024). Thus, Ach signaling plays critical roles in SVZ and SGZ NPCs.

What is the mechanism of Ach‐mediated NPC biology regulation? Optogenetic activation of cholinergic neurons in the SVZ, but not the striatum, results in Ach release and inward currents in SVZ NPCs via the M1 muscarinic receptor expressed in NPCs (Paez‐Gonzalez et al., 2014). In vitro, exogenous Ach stimulation in SVZ NPCs results in phosphorylation of fibroblast growth factor receptor (FGFR), but not epidermal growth factor receptor (EGFR) (Paez‐Gonzalez et al., 2014). Interestingly, Ach did not directly stimulate the FGFR, but instead induced production of FGF ligand by NPCs (Paez‐Gonzalez et al., 2014). Importantly, FGFR phosphorylation and neuroblast formation was reduced when NPCs were incubated with Ach and FGF‐specific function blocking antibody in culture. These results were corroborated in vivo in young (postnatal day [P]30) Ank3 conditional knockout mice on Drd2 background, which displayed reduced SVZ cholinergic neuron firing along with reduced FGFR phosphorylation in SVZ NPCs and defects in neuroblast formation (Paez‐Gonzalez et al., 2014).

While it is clear that local SVZ cholinergic neurons are critical regulators of SVZ NPC fates, it is also known that lesions to the cholinergic basal forebrain reduce OB neurogenesis (Cooper‐Kuhn et al., 2004) (Table 1; Figure 2). While this hints at another important source of cholinergic regulation of SVZ NPCs and/or their progeny in the OB, it is not clear whether this is a direct or indirect effect. For example, cortical neurons can indirectly modulate SVZ NPC fates by participating in neuronal circuits with cholinergic neurons present in the SVZ niche (Naffaa et al., 2023) (please see Section 1.3). In the future, it will be important to map out neuronal networks that include local and/or distant cholinergic neurons and dissect their direct and indirect roles in regulating SVZ NPC fates.

### 1.3 Regulation of NPCs by cortical glutamatergic and GABAergic neurons

Cortical neurons are comprised of excitatory glutamatergic pyramidal and inhibitory GABAergic neurons (interneurons). The newborn excitatory neurons, which are generated by embryonic Pax6+ cortical precursors, migrate radially along the cortical precursor radial processes to populate the cortex in an inside‐out fashion (Gauthier‐Fisher & Miller, 2013). In contrast, newborn interneurons, which are generated by Nkx2.1+ MGE precursors, migrate tangentially into the embryonic cortex via the intermediate zone tracts (Anderson et al., 1997; Marín & Rubenstein, 2001; Yokota et al., 2007). Once they reach the cortex, interneurons can move down into the VZ/SVZ zones, where they interact with cortical precursor cell soma, or radially along the cortical precursor radial processes into the cortical plate, where they establish neuronal networks with excitatory neurons (Nadarajah et al., 2002; Yokota et al., 2007).

Thus, both excitatory and/or inhibitory cortical neurons make contacts with embryonic cortical precursor cell soma located in the VZ/SVZ and/or radial processes throughout the cortical layers. Not surprisingly, both excitatory and inhibitory cortical neurons were shown to regulate cortical progenitor fates (Barnabé‐Heider et al., 2005; Voronova et al., 2017; Yuzwa et al., 2016) (Table 1; Figure 1). Co‐cultures of cortical precursors with cortical excitatory neurons reduce NPC proliferation and enhance neurogenesis, whereas co‐cultures with inhibitory neurons increase NPC proliferation as well as oligodendrogenesis (Voronova et al., 2017; Yuzwa et al., 2016). Moreover, media conditioned by cortical excitatory neurons also promotes astrogliogenesis (Barnabé‐Heider et al., 2005). These effects on NPC behavior are summarized in Table 1 and Figure 2. While these studies did not investigate whether the effect of cortical neurons is activity‐dependent, they all showed that the specific effects on cortical precursor cell fates are mediated by neurochemicals secreted by neurons. These effects on NPC behavior are summarized in Table 1 and Figure 2.

To determine the identity of the secreted neurochemicals responsible for the observed effects on cortical precursor cell fates, studies employed either a previous literature search (Barnabé‐Heider et al., 2005) or a computational modeling approach (Voronova et al., 2017; Yuzwa et al., 2016). The latter utilized microarray datasets obtained from E13 cultured cortical precursors or embryonic cortical excitatory or inhibitory neurons (Voronova et al., 2017; Yuzwa et al., 2016). These data were used to generate a transcriptome‐based network that predicted neurochemicals that could act on cognate receptors expressed in cortical progenitors (Voronova et al., 2017; Yuzwa et al., 2016). Yuzwa et al. has further bolstered this modeling paradigm by performing mass‐spectrometry of the glycosylated membrane bound proteins of cortical neurons and precursors (Yuzwa et al., 2016). These approaches resulted in identification of a myriad of neurochemicals secreted by cortical excitatory or inhibitory neurons that were predicted to act on cortical progenitors (Barnabé‐Heider et al., 2005; Voronova et al., 2017; Yuzwa et al., 2016). Select ligands were then systematically screened to determine which can recapitulate the effects observed with neuron‐conditioned media.

With regard to the pro‐neurogenic factors secreted by cortical excitatory neurons, glial‐derived neurotropic factor (GDNF), interferon gamma (IFNγ), macrophage inflammatory protein 1β (Mip1β), cardiotrophin like cytokine factor 1 (CLCF1), and neuropeptide Y (NPY) increased cortical progenitor neurogenesis and reduced cortical progenitor proliferation in vitro (Yuzwa et al., 2016) (Figure 1). Furthermore, injection of a cocktail of these ligands directly into the E13 brain lateral ventricles resulted in a significant increase in Tbr2+ intermediate progenitors and Satb2+ newborn neurons in E15 cortex in vivo. Injected embryos also had a reduction in the proportion of proliferating embryonic cortical progenitors. Interestingly, only IFNγ promoted astrogliogenesis, and all factors but Mip1β reduced oligodendrogenesis from cortical progenitors in culture (Figures 1 and 2). Culturing embryonic cortical progenitors in cortical neuron conditioned media with function blocking antibodies against GDNF, neurturin (Nrtn), IFNγ, Mip1β, and an NPY receptor antagonist negated the previously observed effect on increased neurogenesis, confirming these ligands mediated cortical neuron pro‐neurogenic effects. Finally, knockdown of the IFNγ receptor in embryonic cortical progenitors in vivo via in utero electroporation resulted in reduced neurogenesis, further confirming the critical role of IFNγ in embryonic neurogenesis (Yuzwa et al., 2016).

Cultured embryonic cortical neurons also express ciliary neurotrophic factor (CNTF), leukemia inhibitory factor (LIF), and cardiotropin‐1 (CT‐1) (Barnabé‐Heider et al., 2005) (Figure 1), known gliogenic factors that activate the gp130‐JAK–STAT pathway (Bonni et al., 1997; Ochiai et al., 2001; Rajan & McKay, 1998). When added to cortical progenitor cultures, these promote astrogliogenesis, thus recapitulating cortical neuron conditioned media effects (Barnabé‐Heider et al., 2005). Overexpression of CNTF in E15 embryonic cortical progenitors in vivo increases astrogliogenesis, as evidenced by increased S100β staining in the resulting E18 embryonic cortex. However, CNTF was not confirmed to be expressed by cortical neurons in vivo. Instead, the authors show CT‐1 is both expressed and secreted by NeuN+ neurons of the embryonic cortex (Barnabé‐Heider et al., 2005). Cortical progenitors cultured from CT‐1^−/−^ brains show an overall decrease in astrocyte number, and in vivo analysis of the knockout cortices found reduced GFAP and CD44 protein expression at P0/1, with a persistent decrease in GFAP staining at P7. Finally, cortical progenitors cultured in cortical neuron conditioned media with function blocking antibodies for CT‐1, but not LIF, abolished increased astrocyte generation (Barnabé‐Heider et al., 2005). The authors further showed that the media conditioned by cortical neurons activated the gp130‐JAK–STAT pathway in embryonic cortical progenitors (Barnabé‐Heider et al., 2005). Inhibition of this pathway with AG490, a JAK inhibitor, or knockdown of STAT3 in cortical progenitors reduced astrogliogenesis in the presence of conditioned media in vitro (Barnabé‐Heider et al., 2005). Finally, knockdown of gp130 in E14/15 cortical progenitors in vivo led to a reduction in cortical astrogliogenesis at P3/4 (Barnabé‐Heider et al., 2005). gp130 is a receptor required for all cytokine signaling (Bonni et al., 1997; Ochiai et al., 2001; Taga & Kishimoto, 1997). The requirement of gp130 by embryonic cortical progenitors for astrogliogenesis suggests that astrocyte formation is primarily mediated by cytokines. Overall, embryonic cortical neurons were found to regulate embryonic astrogliogenesis via CT‐1 and regulation of the JAK–STAT pathway (Barnabé‐Heider et al., 2005) (Table 1; Figure 1).

Inhibitory cortical neurons express and secrete growth arrest specific 6 (Gas6) and fractalkine (FKN/CX3CL1) (Tarozzo et al., 2003; Voronova et al., 2017; Watson et al., 2020) (Figure 1). These neurochemicals both showed a pro‐oligodendrogenic effect when added to embryonic cortical progenitor cultures in vitro (Voronova et al., 2017). However, while Gas6 promoted the survival of oligodendrocytes, FKN promoted commitment of NPCs to OPCs, and differentiation of OPCs into oligodendrocytes. Notably, both Gas6 and FKN do not affect precursor proliferation, neurogenesis or astrogliogenesis (Voronova et al., 2017). Furthermore, interneurons were found to be a major source of FKN in vivo (Voronova et al., 2017). Ablation of Nkx2.1+ MGE progenitors and their derivatives interneurons in vivo (*Nkx2.1Cre;Rosa26* ^*STOP‐DTA*^) reduces total and proliferating cortical OPCs, as well as cells expressing *Fkn* mRNA in the E18 cortical VZ/SVZ niches (Voronova et al., 2017). As these mice are perinatal lethal, the analysis of the postnatal cortical development was not possible. Finally, single‐molecule fluorescent in situ hybridization (FISH; RNA scope) of the embryonic and postnatal cortex identified FKN receptor *Cx3cr1* expression in NPCs, OPCs and microglia. Mice with constitutive *Cx3cr1* knockout or knockdown in embryonic cortical progenitors and their progeny showed reduced OPC and oligodendrocyte formation in vivo (Voronova et al., 2017). The ability of FKN to promote oligodendrogenesis from NPCs and OPCs was later demonstrated in postnatal cerebellar ex vivo slices as well as in vivo in adult healthy and demyelinated murine brain (de Almeida et al., 2023; Watson et al., 2021). Thus, the ability of developmentally important neurochemicals to modulate NPC fates may be preserved throughout the lifespan and in injury, making studies of neuron‐NPC communication a powerful platform for pro‐regenerative molecule discovery.

Collectively, the reports cited above establish a model for the temporal control of embryonic cortical progenitor cell fates. Cortical precursor differentiation is under a strict temporal control, where neurons are generated first, followed by astrocytes and then oligodendrocytes (Adnani et al., 2018; Gauthier‐Fisher & Miller, 2013). One potential explanation for this pattern of differentiation is the rapidly changing developing cortex milieu in which cortical progenitors bathe in. The reports mentioned above establish a putative model, where cortical precursors first expand their pool and secrete pro‐proliferating factors, followed by the formation of excitatory neurons, which further promote neurogenesis at least in part via IFNγ (Yuzwa et al., 2016). At a later time, or once the critical mass of excitatory neurons is formed, these neurons then induce astrogliogenesis via CT‐1 (Barnabé‐Heider et al., 2005). Finally, interneurons populate the cortex during late embryogenesis and promote oligodendrogenesis at least in part via FKN (Voronova et al., 2017).

In postnatal development and adulthood, cortical afferents regulate SVZ NPC fates via direct innervation of the SVZ niche as well as indirectly by regulating the activity of the local SVZ niche neurons (Table 1). In juvenile mice (P21), cortical neuron axons innervate the dorsolateral, lateral, and septal areas of the SVZ niche and make contacts with Dcx + cells (Ota et al., 2023). A stab injury to the postnatal medial cortex increases total Dcx + neuroblasts in the SVZ and proliferating oligodendrocyte transcription factor 2 (Olig2) + oligodendroglial cells in the striatum (Ota et al., 2023). In adulthood, anterior cingulate cortical (ACC) glutamatergic afferents modulate SVZ NPC fates by participating in a neural circuit involving ChAT+ axons located in the SVZ niche (Naffaa et al., 2023). As mentioned above, ChAT+ axons are known to directly regulate SVZ NPC fates (Paez‐Gonzalez et al., 2014) (see Section 1.2). Activation of the ACC glutamatergic inputs activates the SVZ ChAT+ neurons and concomitantly increases ventral SVZ NPC proliferation and commitment to Dcx + neuroblasts (Naffaa et al., 2023). Notably, optogenetic silencing of ChAT+ neurons while activating ACC inputs abrogates these changes in the SVZ (Naffaa et al., 2023). In agreement, inhibition of ACC glutamatergic inputs results in inhibition of SVZ ChAT+ neurons and a concomitant decrease in ventral SVZ NPC proliferation and commitment to Dcx + neuroblasts (Naffaa et al., 2023) (Table 1; Figure 2). While the role of the ACC was not tested in the dorsal or lateral SVZ, it was demonstrated that ventral SVZ NPC fates can be modulated indirectly by ACC neuronal axons forming a neuronal circuit with the local SVZ cholinergic neurons (Naffaa et al., 2023). While Ota and colleagues show that dorsolateral SVZ NPC fates may be directly modulated by cortical afferents, the mechanism behind this direct cortical axonal control of the SVZ niche remains to be addressed (Ota et al., 2023).

### 1.4 Regulation of NPCs by substantia nigra and VTA dopaminergic neurons

The dopaminergic (DA) neuron population in the CNS are heterogeneous, located in both the midbrain and diencephalon. These cells express tyrosine hydroxylase (TH), which catalyzes the conversion of l‐tyrosine to l‐3,4‐dihydroxyphenyalanine (l‐DOPA), the rate limiting step in DA formation. Midbrain DA neurons are most studied for their role in the nigrostriatal system, in which cells from the substantia nigra (SNc) regulate motor behavior through projections to the caudate and putamen nuclei of the basal ganglia (Bentivoglio & Morelli, 2005). Degeneration of these cells is the major pathological mechanism in Parkinson's disease (PD) (Beitz, 2014). In addition to the SNc, DA neurons are also found in the ventral tegmental area (VTA), where they largely project into the nucleus accumbens and regulate emotion, motivation, and reward behaviors (Bromberg‐Martin et al., 2010). VTA neurons have also been found to project to the septum, amygdala, and hippocampus (Bentivoglio & Morelli, 2005).

Several groups have reported the presence of TH+ and dopamine transporter (DAT) + axons in the rodent and/or primate SVZ and SGZ niches (Baker et al., 2004; Freundlieb et al., 2006; Höglinger et al., 2004, 2014). These axons lack noradrenergic markers, further confirming their dopaminergic identity. In the SVZ, dopaminergic fibers make contacts primarily with EGFR+ TAPs, some NPCs, but not neuroblasts (Höglinger et al., 2004).

Originally, TH + DAT+ dopaminergic axons in the SVZ were thought to arise specifically from the SNc. The evidence for this observation came from lesion experiments. Dopaminergic neuron death was induced by unilateral injection of 6‐hydroxydopamine (6‐OHDA) into the rodent SNc (Baker et al., 2004; Höglinger et al., 2004). Lesioned animals showed decreased cell proliferation in the SVZ when compared to sham‐injected brain or contralateral SVZ in the lesioned brain (Baker et al., 2004; Höglinger et al., 2004; Sui et al., 2012). Furthermore, the extent of SVZ denervation correlated with the extent of reduction in SVZ cell proliferation (Baker et al., 2004; Höglinger et al., 2004). Interestingly, following 6‐OHDA lesioning, an increase in surviving newborn OB neurons was observed (Sui et al., 2012). As 6‐OHDA represents permanent dopaminergic neurodegeneration, the next step involved a transient SVZ denervation to corroborate these results. Mice or primates were systemically injected with 1‐methyl‐4‐phenyl‐1,2,3,6‐tetrahydropyridine (MPTP), a neurotoxin that degenerates dopaminergic axons but does not ablate SNc cell bodies, therefore allowing for gradual axonal recovery (Freundlieb et al., 2006; Höglinger et al., 2004). In agreement with 6‐OHDA results, MPTP‐mediated denervation of the SVZ resulted in a reduction in proliferative markers in the SVZ, as labeled by proliferating cell nuclear antigen (PCNA) (Freundlieb et al., 2006; Höglinger et al., 2004). A similar result was also seen in the murine SGZ (Höglinger et al., 2004). Mice treated with MPTP showed specific reduction in proliferating EGFR+ cells in the SVZ without affecting total number of cells, or cell death. Notably, the reduction in proliferation was accompanied by a concomitant reduction in neuroblasts, which are specified from EGFR+ TAPs (Höglinger et al., 2004).

The idea that all dopaminergic axons in the SVZ originate from SNc was challenged by Lennington and colleagues (Lennington et al., 2011). These authors hypothesized that some of the dopaminergic axons in the SVZ may originate from the VTA, which is adjacent to the SNc. To this end, the *aphakia* mouse model was used, which carries *Pitx3* gene variant, leading to a selective loss of the SNc dopaminergic neurons during embryonic development persisting into adulthood (Hwang et al., 2003; Nunes et al., 2003). Despite the profound loss of dopaminergic fibers in the striatum, *aphakia* mice show normal TH+ axonal innervation of the SVZ niche as well as unaltered SVZ organization and SVZ cell fates when compared to control mice (Lennington et al., 2011). The authors then sought to determine what would happen to the SVZ if the VTA dopaminergic neurons were also ablated. First, they showed that intraperitoneal MPTP injections targeted both SNc and VTA dopaminergic neurons in the wild‐type (WT) and VTA neurons in the SNc neuron‐deficient *aphakia* mice. In agreement with (Höglinger et al., 2004), MPTP injections significantly reduced proliferation in the SVZ in WT mice (Lennington et al., 2011). Importantly, MPTP injections also reduced SVZ cell proliferation in *aphakia* mice at comparable levels to MPTP‐mediated proliferation reduction in WT mice (Lennington et al., 2011). While these results are compelling, it is possible that VTA innervation of the SVZ could be a compensatory mechanism in *aphakia* mouse model. This is in part supported by the observation that in WT mice, TH + DAT+ axonal innervation of the striatum and adjacent SVZ begins after E14.5 and increases thereafter (Fauser et al., 2020). In *aphakia* mice, SNc neurons die before birth (Hwang et al., 2003). Therefore, it is possible that VTA innervation of the SVZ in *aphakia* mice may be formed or remodeled differently during embryogenesis and *aphakia* mice may not represent normal dopaminergic axonal innervation present in the WT mice.

To this end, Lennington and colleagues performed both retrograde and anterograde tracing in both WT and *aphakia* mice, confirming the VTA does project into the SVZ (Lennington et al., 2011). This was corroborated and extended by Höglinger and colleagues a few years later. Retrograde tracing in the dorsal rat SVZ labeled neurons in the SNc, while retrograde tracing in the ventral rat SVZ labeled neurons in the VTA (Höglinger et al., 2014). This deviates from Lennington et al., which found the VTA projects into both dorsal and ventral SVZ (Figure 1). The discrepancies can be attributed to the surgeon's skills and/or animal species (mice vs. rats) used. Nevertheless, both studies confirmed VTA dopaminergic neurons project into the SVZ. Lastly, Höglinger and colleagues performed in‐depth spatially resolved anterograde tracing via injections of DiI into the rat VTA or different SNc regions (Höglinger et al., 2014). Injections into the VTA identified dopaminergic axons specifically in the ventral SVZ as well as SGZ. Injection of DiI into the anterior SNc resulted in identification of TH + DiI+ axons in the rostral SVZ, near the RMS. The medial SNc neurons projected to both the RMS and ventromedial SVZ, while the lateral SNc projected to the dorsolateral SVZ. Finally, the posterior SNc was found to project to the dorsal SVZ (Höglinger et al., 2014). Therefore, both SNc and VTA dopaminergic neurons project into the SVZ in a spatial manner.

Regardless of the source of the dopaminergic axonal innervation, 6‐OHDA and MPTP injections into WT mice show that collectively dopaminergic axons regulate SVZ cell proliferation (Baker et al., 2004; Freundlieb et al., 2006; Höglinger et al., 2004; Lennington et al., 2011) (Table 1; Figure 2). Does dopaminergic denervation have long‐lasting consequences on adult neurogenesis? Transient dopaminergic axonal denervation via MPTP systemic injections or permanent denervation via 6‐OHDA SNc injections results in altered OB neurogenesis (Höglinger et al., 2004; Winner et al., 2006) (Table 1; Figure 2). While Höglinger and colleagues found an overall decreased number of newborn neurons in the murine OB after MPTP treatment (Höglinger et al., 2004), spatially resolved analysis of rat OB neurogenesis by Winner and colleagues revealed a dichotomous response to 6‐OHDA‐mediated dopaminergic innervation (Winner et al., 2006). Namely, Winner and colleagues found a transient decrease in newborn neurons in the rat OB granule cell layer 14 days post‐6‐OHDA injections, and a persistent increase in newborn neurons in the OB glomerular layer at 42 days post‐6‐OHDA injections (Winner et al., 2006). The difference in the results could be because of animal species (mouse vs. rat), sex (male mice vs. female rats), or nature of dopaminergic denervation (transient vs. permanent). Nevertheless, the conclusion that dopaminergic innervation controls SVZ NPC proliferation and OB neurogenesis is well‐supported by the evidence in both mice and rats and may have a significant clinical relevance. Namely, postmortem analysis of brains from patients diagnosed with Parkinson's disease (PD), which is characterized by dopaminergic neurodegeneration, reveal reduced PCNA staining (proxy of reduced proliferation) in the SVZ compared to subjects that did not have a history of a neuropsychiatric disorder (Höglinger et al., 2004). Moreover, PD OBs have fewer Nestin+PSA‐NCAM+ immature neurons or neuroblasts (Höglinger et al., 2004). Interestingly, aberrant olfaction is detected in both symptomatic and pre‐symptomatic (prodromal) stages of PD (Berendse et al., 2001). Together, these studies demonstrate that dopaminergic axons regulate SVZ cell proliferation and OB neurogenesis and function in rodents, primates, and humans (Baker et al., 2004; Freundlieb et al., 2006; Höglinger et al., 2004, 2014).

There are several proposed mechanisms for how dopaminergic axons may regulate SVZ cell fates. First, electron microscopy analysis of ultrathin rodent SVZ sections revealed the presence of synaptic vesicles at the dopaminergic axonal terminals, which in turn made contacts with SVZ TAPs (Höglinger et al., 2004). This suggests that the axon‐TAP communication may be mediated through a neurotransmitter release. Both immunohistochemistry and electron microscopy show the dopamine family D2‐like (D2L) receptor is present on multiple cells in the SVZ, including TAPs and some neuroblasts. Cultured SVZ neurosphere cells increase proliferation in response to treatment with D2L receptor agonist bromocriptine (Höglinger et al., 2004). While D2L receptor antagonist sulpiride does not alter SVZ neurosphere cell proliferation, it blocks bromocriptine‐enhanced proliferation of these cells. Notably, D1‐like receptor modulators do not have an effect (Höglinger et al., 2004). Therefore, activation of D2L receptor is sufficient to induce enhanced NPC proliferation in vitro. In vivo, reduced SVZ proliferation in 6‐OHDA injected rat brains was rescued with subcutaneous delivery of levodopa, the dopamine precursor molecule. Furthermore, administration of a selective D2L agonist ropinirole increased proliferation in the SVZ of both lesioned and nonlesioned rat brains (Höglinger et al., 2004). The importance of the D2L receptor signaling was confirmed by another group. First, they showed that quinpirole, a D2 receptor agonist treatment of adult naïve mice increased BrdU+ and Dcx + cells in the SVZ (Peng et al., 2008). Furthermore, the authors showed that this effect was absent in mice that lacked CNTF by using CNTF knockout mice or mice infused with CNTF‐function blocking antibodies (Peng et al., 2008). This suggests that D2L‐mediated SVZ cell fate regulation is at least in part dependent on CNTF. The authors further showed that dopaminergic denervation via 6‐OHDA injections reduces CNTF expression, while D2 stimulation via quinpirole increases CNTF expression in SVZ astrocytes. It is not clear whether the parenchymal GFAP+ astrocytes or GFAP+ SVZ NPCs as the source of CNTF. Finally, Winner and colleagues observed that dopaminergic denervation in adult female rats following 6‐ODHA lesions of the SNc increases the numbers of Pax6+ cells in the SVZ (Winner et al., 2006). While Pax6 regulates OB TH+ neuron differentiation in embryogenesis (Dellovade et al., 1998), the significance of this finding in adult SVZ niche remains to be addressed.

Overall, the reports discussed above demonstrate a critical role of the dopaminergic innervation of the SVZ niche in regulating NPC fates (Figure 2). In future, it will be important to extend these findings beyond lesioning and tracing techniques, such as opto‐ or chemogenetic approaches, to probe the role of the innervating dopaminergic axonal activity in SVZ NPC biology.

### 1.5 Regulation of NPCs by hypothalamic neuropeptidergic neurons

The hypothalamus is a collection of brain nuclei comprised of glutamatergic and GABAergic neurons that project widely throughout the brain and exert overlapping and distinct functions, including feeding behavior (Pasquier & Reinoso‐Suarez, 1978; Sebastien et al., 2004). The arcuate nucleus, a hypothalamic nucleus that regulates feeding behaviors, innervates the SVZ (Arias‐Carrion et al., 2018; Paul et al., 2017), while the supramamillary nucleus (SuM), a hypothalamic nucleus that controls reward‐seeking, exploration and social memory, innervates the SGZ (Kesner et al., 2023) (Table 1; Figure 1). Notably, the hypothalamus was recently discovered to have its own neurogenic niche, which has been reviewed elsewhere (Bartkowska et al., 2023; Sharif et al., 2021).

The arcuate nucleus contains two populations of neuropeptidergic neurons; the anorexigenic pro‐opiomelanocortin (POMC) and orexigenic neuropeptide Y/Arginine related peptide (NPY/AgRP) neurons (Saper et al., 2002). Anorexigenic POMC+ afferents innervate the ventral SVZ while orexigenic NPY/AgRP afferents innervate the dorsal SVZ (Arias‐Carrion et al., 2018; Paul et al., 2017). Chemogenetic activation of POMC+ neurons increases, while ablation of POMC+ neurons through injection of an AAV expressing cleaved caspase 3 (AAV‐Flex‐Casp3) into *Pomc‐Cre* mice reduces ventral SVZ NPC proliferation (Paul et al., 2017). With regard to NPY/AgRP+ neurons, their pharmacological ablation through injection of orexin‐SAP, a neurotoxin, directly into the hypothalamus of adult male rats, increases cell proliferation in the SVZ (Arias‐Carrion et al., 2018). Notably, this result is opposite of the anorexigenic denervation reported by Paul and colleagues (Paul et al., 2017). It is possible that the fates of dorsal and ventral SVZ NPCs are governed at least in part because of the nature of hypothalamic afferents present in the SVZ niche regions, with anorexigenic POMC+ neuronal axons innervating and regulating ventral SVZ and orexigenic NPY/AgRP fibers innervating and regulating dorsal SVZ. While the mechanism of orexigenic NPY/AgRP fibers interaction with dorsal SVZ NPCs is not currently known, application of β‐endorphin, an endogenous opioid produced by anorexigenic POMC+ neurons (Richard et al., 2001), increases activation and proliferation of Nestin+ SVZ NPCs in culture and Nkx2.1+ NPCs in the ventral SVZ in vivo (Paul et al., 2017). Thus, β‐endorphin mimics the effect of POMC+ neuron activation on ventral SVZ NPCs.

Given the existing body of work connecting POMC+ neurons with feeding behavior (Aponte et al., 2011; Zeltser et al., 2012), Paul and colleagues assessed how changes in feeding behavior affect SVZ NPCs fates via the POMC neuron activity (Paul et al., 2017). Adult NPC reporter mice were either fed ad libitum, fasted or fasted and refed. Fasting reduced activity of POMC neurons and NPC proliferation specifically in the ventral SVZ (Paul et al., 2017). This reduction in hypothalamic neuron activity and proliferating SVZ NPCs was reversed when animals were refed, as well as when POMC+ neurons were activated via DREADDs in fasted animals (Paul et al., 2017). Together, these results demonstrate a direct effect of hunger and satiety on ventral SVZ NPCs via hypothalamic neuronal activity. These neuronal‐dependent changes in the SVZ were further demonstrated to be long‐lasting (Paul et al., 2017). Following either fasting or POMC neuron ablation in mice fed ad libitum, the number of newborn neurons in the OB was reduced (Paul et al., 2017). This confirms that hypothalamic neuronal activity regulates not only local SVZ NPCs within the vicinity of the innervating hypothalamic axons, but also has downstream long‐lasting effects on neurogenesis in the OB.

The hypothalamic SuM nucleus is comprised of glutamatergic and GABAergic neurons, which project into the dentate gyrus of the hippocampus and modulate adult SGZ NPC neurogenesis (Li et al., 2022). Activity of SuM neurons is required for early hippocampal neurogenesis. Short‐term optogenetic and chemogenetic stimulation of SuM neurons in vivo (for 3 days) increases proliferation of both Nestin+ NPCs and Dcx + neuroblasts (Li et al., 2022). In agreement, chemogenetic inhibition of SuM neurons reduces NPC and neuroblast proliferation (Li et al., 2022). With regard to neurochemical mechanism, the SuM stimulates SGZ NPC proliferation specifically via glutamatergic AMPA and NMDA, and not GABAergic, receptors (Li et al., 2022). In contrast, the sustained effects on long‐term NPC neurogenesis requires SuM GABAergic neurons and neurochemical modulation of newborn immature neurons (Li et al., 2022). To this end, adult‐born immature neurons were found to receive direct monosynaptic GABAergic, but not glutamatergic inputs (Li et al., 2022). Furthermore, 16‐day stimulation of SuM neurons promotes dendritic development in newly born immature neurons, while chronic stimulation of SuM neurons for 32 days increases the proportion of newborn NeuN+, and decreases Dcx + cells, therefore confirming SuM neurons promote maturation of neurons. Overall, the authors concluded that while SuM glutamatergic inputs directly promote SGZ NPC proliferation and early neurogenesis, SuM GABAergic inputs promote late neurogenesis by acting on immature neurons (Li et al., 2022). Notably, adult born hippocampal neurons modulate memory formation and emotional behaviors (Anacker & Hen, 2017; Kumar et al., 2020; Lods et al., 2021; Tunc‐Ozcan et al., 2019; Wang et al., 2021). In agreement, modulation of SuM neuron activity exhibits changes in both spatial and contextual memory formation as well as anxiety like but not depressive behavior, presumably via modulation of hippocampal neurogenesis (Li et al., 2022).

Collectively, the studies reviewed above provide compelling evidence for regulation of SVZ and SGZ NPC fates via hypothalamic innervation. Within the SVZ, different hypothalamic neurons innervate distinct SVZ regions with opposing effects on NPC proliferation. Finally, hypothalamic innervation in both SVZ and SGZ has long‐term behavioral consequences. In the future, it will be important to determine additional contributions of hypothalamic nuclei to adult neuro‐ and gliogenesis in health and disease.

### 1.6 Regulation of NPCs by raphe nuclei serotonergic neurons

The raphe nuclei are located within the brain medulla and are the major source of serotonergic neurons projecting throughout the forebrain. Serotonergic innervation of the adult SVZ was first noted in Aghajanian and Gallager (1975), who lesioned the dorsal and pontine raphe nuclei of male albino rats with an electrothermic electrode and observed a reduction in neuronal projections in the subependymal zones of the brain ventricles. Raphe projections into the SVZ were corroborated by several other groups (Lorez & Richards, 1982; Mathew, 1999). More recent work elegantly mapped out the plexus of raphe fiber projections within the SVZ (Tong et al., 2014) (Table 1; Figure 1). Retrograde tracing via fluorescent retrobead injection into the ventricles coupled with wholemount adult murine SVZ preparations revealed a dense plexus of unmyelinated axons originating in the dorsal raphe. This was corroborated with anterograde tracing by injecting biotinylated dextran amide into the dorsal raphe, which was later detected in the axons within the SVZ (Tong et al., 2014). Immunostaining revealed NPCs present throughout the entire SVZ were often associated with axons expressing serotonin, while transmission electron microscopy showed these axons contacted NPCs and were wrapped by NPC microvilli. Interestingly, despite this contact, NPCs were not seen to have postsynaptic structures, but did have membrane invaginations and clathrin coated pits, suggesting axon‐NPC communication does occur, but may not be synaptically mediated (Tong et al., 2014). However, an injection of an anterograde vesicular stomatitis virus expressing EGFP, which spreads transsynaptically, into the dorsal raphe nuclei resulted in robust EGFP labelling of NPCs and neuroblasts in the SVZ and RMS four days post injection suggesting there may be synaptic communication between NPCs and neurons despite ultrastructural analysis not showing post‐synaptic structures on NPCs (Tong et al., 2014).

Regulation of adult SVZ neurogenesis by raphe projections is evidenced by chemical lesion approaches (Brezun & Daszuta, 1999) (Table 1). Injection of 5,7‐dihyroxytryptamine (5,7‐DHT), a neurotoxin that specifically ablates serotonergic and noradrenergic neurons when metabolized by monoamine oxidases (MAO) (Finnegan et al., 1989), directly into the raphe nuclei, or intraperitoneal injections of parachlorophenylalanine (PCPA), which inhibits serotonin synthesis without degenerating neurons (Koe & Albert, 1966), result in a decrease in serotonergic nerve terminals and number of BrdU+ cells and PSA‐NCAM+ neuroblasts in the SVZ and SGZ (Brezun & Daszuta, 1999). This was corroborated with intracerebroventricular infusion of fenfluramine, which promotes the release of serotonin (Tong et al., 2014). Fenfluramine‐infused mice had increased number of BrdU+ cells and Dcx + cells in the SVZ compared to saline infused animals, supporting the effect of serotonin on proliferation and neurogenesis (Tong et al., 2014). Moreover, transplanted fetal raphe, but not spinal cord, neurons secrete serotonin and rescue deficient SGZ NPC proliferation in postlesioned PCPA treated mice (Brezun & Daszuta, 2000). Thus, serotonergic signaling from raphe neuron projections regulates SVZ and SGZ NPC proliferation and/or neurogenesis (Figure 2).

Serotonin is well known to have widespread effects throughout the brain and has a large range of receptors (Banasr et al., 2004; Tierney, 2018). Fluorescent‐activated cell sorting (FACS) of adult SVZ NPCs microdissected from NPC reporter mice revealed that only 5HT2C and 5HT5A receptors were expressed by SVZ cells (Tong et al., 2014). Application of serotonin to adult murine SVZ wholemount preparations results in inward currents in SVZ NPCs likely mediated by potassium ion currents. Application of specific agonists and antagonists for the 5HT2C and 5HT5A receptors to the wholemount SVZ preparations induced and abolished this current, respectively. Furthermore, intracerebroventricular infusion with a selective 5HT2C agonist in adult mice for either 4 h or 5 days increased the number of BrdU+ proliferating SVZ NPCs, without affecting neuroblasts, Mash1+ IPs or total number of SVZ cells (Tong et al., 2014). In agreement, infusion of 5HT2C antagonist had the opposite effect (Tong et al., 2014). These findings align with previous work that identified 5HT2A and 5HT2C regulate adult NPC proliferation (Banasr et al., 2004). Therefore, SVZ NPCs respond to serotonin signaling by modulating proliferation and neurogenesis.

The studies discussed above elegantly demonstrate that serotonergic innervation by raphe nuclei is important for SVZ NPC proliferation and neurogenesis (Figure 2). However, as raphe is a collection of nuclei, the contributions of each nucleus to SVZ NPC regulation as well as the mechanism of serotonergic regulation of NPC fates (e.g., via neuronal activity) remain to be addressed.

### 1.7 Regulation of NPCs by locus coeruleus noradrenergic neurons

The main noradrenergic (NA) nucleus of the brain is the locus coeruleus (LC), or “*blue spot*,” located in the pons. LC neurons project into the OB, and their degeneration results in OB dysfunction (Guérin et al., 2009; McLean et al. 1989; Shipley et al., 1985). Furthermore, stimulation of NA release in adult mice by α2 receptor antagonist dexefaroxan reduces apoptotic cells in the SVZ and OB, as well as increases the number of proliferating cells in these regions in both healthy and OB axotomized animals (Bauer et al., 2003; Veyrac et al., 2005). Noradrenergic signaling also modulates OB‐related behaviors in mice, rats, and sheep (Brennan et al., 1998; Levy, 1990; Sullivan et al., 1992). Finally, addition of noradrenaline to SVZ neurosphere cells increases neurogenesis in vitro (Kärkkäinen et al., 2009) (Table 1; Figures 1 and 2).

The role of LC innervation in the embryonic SVZ was demonstrated in rat brain explants (Popovik & Haynes, 2000). The rostral pons, containing the LC, was dissected from E15 rat embryos and arranged near explants from E13 neocortex and cultured for 10 days. Retrograde labeling identified dopamine β‐hydroxylase‐positive neurons from the pons explant projecting to the neocortex tissue, and transection of these fibers led to permanent cortical tissue noradrenergic denervation and increase in cortical cell apoptosis (Popovik & Haynes, 2000). The observed changes in cell apoptosis were specific to LC noradrenergic innervation. For example, coculturing diagonal band/septum explants that contain *cholinergic* neurons (see Section 1.2) with E13 cortex led to *cholinergic* innervation of the cortex. However, cholinergic projection transection did not change the number of cortical apoptotic cells. Finally, noradrenergic afferents also regulate embryonic NPC proliferation. Addition of noradrenergic receptor α1, but not α2, receptor antagonists to the LC‐cortex cocultures reduce proliferating cortical cells (Popovik & Haynes, 2000). The role of the α1 receptor in NPC proliferation was later corroborated in the embryonic and adult hippocampus in vivo (Gupta et al., 2009; Kulkarni et al., 2002), and in vitro (Gupta et al., 2009; Hiramoto et al., 2006). Thus, LC innervation regulates embryonic cortical apoptosis and proliferation ex vivo. Whether LC neurons innervate and regulate embryonic or adult SVZ NPCs in vivo remains to be addressed.

### 1.8 Discussion and future directions

Communication of NPCs with neighboring neurons and/or their axons is emerging as a critical regulator of SVZ and SGZ NPC survival, proliferation, and differentiation (Table 1; Figures 1 and 2). In contrast to OPCs, which form bona fide synapses with neurons (Maas & Angulo, 2021; Moura et al., 2022; Paukert & Bergles, 2006; Taylor & Monje, 2023), the majority of reports discussed above do not support a synaptic connection between NPCs and neurons. Instead, the reports highlight the neurotrophic effect of neurotransmitters and neurochemicals on NPC fates. Below, we discuss the existing knowledge gaps and potential future directions in this field.

First, embryonic cortical neurons were only evaluated for trophic release of neurochemicals and not activity‐dependent control of embryonic SVZ NPCs. However, a recent report demonstrates embryonic neurons are electrically active during early embryogenesis (Munz et al., 2023). This raises the possibility that some of the embryonic NPC fates may be mediated by embryonic neuronal activity. On the other hand, adult neurons were only evaluated for activity‐dependent control of SVZ and SGZ NPCs. Yet, neurotransmitters like dopamine and GABA can signal through both electrochemical and tonic actions (Dreyer et al., 2010; Farrant & Nusser, 2005; Grace, 1991). As discussed above, GABA from both tonic and phasic release can modulate NPC fates (Liu et al., 2005; Young et al., 2014). This raises the possibility that adult neurons may secrete neurotransmitters or other neurochemicals tonically, which may have implications on the neighboring NPCs.

Second, while the adult SVZ niche is heterogeneous with dorsal, lateral, ventral, and septal NPCs participating in distinct progeny generation (e.g., various OB interneurons vs. glia (Chaker et al., 2016; Obernier & Alvarez‐Buylla, 2019)), most of the reports identifying neuronal innervation in the SVZ niche do not account for this spatial heterogeneity. For example, hypothalamic POMC+ neurons specifically innervate the ventral SVZ (Paul et al., 2017), while NPY/ArGP neurons project to the dorsal SVZ (Arias‐Carrion et al., 2018). Importantly, POMC+ and NPY/ArGP neurons have opposing effects on ventral and dorsal SVZ NPC proliferation, respectively (Arias‐Carrion et al., 2018; Paul et al., 2017). Given the heterogenous nature of adult SVZ NPCs, identifying which regional NPC populations are regulated by which neuronal projections will be of interest in future works.

Third, while reports discussed above have elegantly demonstrated the role of several innervating neurons on neurogenesis, only four reports (Barnabé‐Heider et al., 2005; He et al., 2016; Voronova et al., 2017; Yuzwa et al., 2016) addressed the role of neurons in other NPC cell fates, such as astrogliogenesis or oligodendrogenesis. Yet, existing literature supports the hypothesis that various innervating neurons may regulate gliogenesis from adult NPCs. For example, orexigenic NPY/AgRP fibers were found in much higher density in the septal SVZ niche (Arias‐Carrion et al., 2018). In turn, septal SVZ is enriched in platelet‐derived growth factor receptor β (PDGFRβ) + NPCs that are highly gliogenic (Delgado et al., 2021). However, the role of hypothalamic innervation on septal SVZ NPCs or their gliogenic potential is currently not known. Furthermore, it is known that cholinergic signaling promotes OPC differentiation into oligodendrocytes in culture (De Angelis et al., 2012). While short‐term optogenetic modulation of SVZ cholinergic neurons did not result in differences in NG2+ OPC number or proliferation (Paez‐Gonzalez et al., 2014), it is known that differentiation of astrocytes and oligodendrocytes in general takes longer than formation of neurons (Contreras et al., 2021; Dittmann et al., 2023; Figueres‐Oñate et al., 2019). In this light, in the future, it will be important to examine the influence of neurons on formation of glial cells from NPCs.

Finally, as changes in neuronal activity impact NPC fates (as discussed above), it would be important to understand how aberrant neuronal connectivity in neurodevelopmental disorders or abnormal neuronal activity in epilepsy affect SVZ and SGZ NPC biology. As elegantly demonstrated by Naffaa and colleagues, cortical neurons are able to indirectly regulate SVZ NPC biology via establishing a neuronal circuitry with cholinergic neurons in the SVZ niche (Naffaa et al., 2023). Thus, changes in seemingly distant neural circuits could have profound and long‐lasting effects on NPCs and associated behaviors. Adult SVZ and SGZ NPC fates are altered in mouse models of neurodevelopmental disorders (Gallagher et al., 2015; Hourigan et al., 2021). As such, it would be important to understand whether these abnormalities are as a result of autonomous roles of disease‐causing gene mutations in NPCs or because of aberrant neuronal innervation of the neurogenic niches or other perturbations of the neuronal circuitry that could directly or indirectly impact the NPC fates via dysfunctional neuron‐NPC communication. The ability of distantly activated neurons to act on NPCs is also important to understand in the context of deep brain stimulation for movement disorders, such as PD, and transcranial neuromodulation for neuropsychiatric disorders such as major depression and obsessive‐compulsive disorders (Briley et al., 2024; Pei et al., 2024; Vicheva et al., 2025). While both stimulation protocols are already in clinical practice, short‐ and long‐term consequences of these stimulations on adult NPC fates and associated behavior and cognition are not currently known.

## AUTHOR CONTRIBUTIONS

**Nicole Leanne Dittmann:** Conceptualization; writing – original draft; writing – review and editing. **Lauren Chen:** Writing – review and editing. **Anastassia Voronova:** Conceptualization; funding acquisition; resources; supervision; writing – original draft; writing – review and editing.

## FUNDING INFORMATION

NLD was supported by the Natural Sciences and Engineering Research Council (NSERC) Postgraduate Scholarship‐Doctoral level and Brad Mates E Drive Studentship in Parkinson's Disease and Movement Disorders. LC was supported by the VP (Academic) Summer Studentship Award from the Faculty of Medicine & Dentistry at the University of Alberta. AV was supported by the Canada Research Chair in Neural Stem Cell Biology and Sloan Research Fellowship in Neuroscience awards. This work was funded by NSERC Discovery Grant awarded to AV.

## CONFLICT OF INTEREST STATEMENT

AV is a Handling Editor for the Journal of Neurochemistry. All other authors declare no competing interests.

### PEER REVIEW

The peer review history for this article is available at https://www.webofscience.com/api/gateway/wos/peer‐review/10.1111/jnc.16287.

## Data Availability

No original data was generated.
